# Comparison for efficacy of general exercises with and without mobilization therapy for the management of adhesive capsulitis of shoulder - An interventional study

**DOI:** 10.12669/pjms.316.7909

**Published:** 2015

**Authors:** Saba Aijaz Ali, Muhammad Khan

**Affiliations:** 1 Saba Aijaz Ali, MASPT. Institute of Physical Medicine & Rehabilitation, Dow University of Health Sciences, Karachi, Pakistan; 2 Muhammad Khan, MSc. PT. Institute of Physical Medicine & Rehabilitation, Dow University of Health Sciences, Karachi, Pakistan

**Keywords:** Adhesive Capsulitis, Frozen shoulder, General exercises, Mobilization therapy

## Abstract

**Objective:**

The aim of this work was to evaluate the effectiveness of exercise with manual therapy and exercise alone in adhesive capsulitis of the shoulder.

**Method:**

This randomized study was conducted at institute of physical medicine and rehabilitation Dow University of Health Sciences, Karachi between January, 2014 and July, 2014. Forty four participant age between 25-40 years were recruited. Twenty two participants were allocated to exercise and manual therapy group and 22 participants were allocated to exercise only group. Exercise and manual therapy group received general exercises and Maitland mobilization on shoulder joint whereas exercise group only received general exercises. Both interventions were carried out 3 times a week for 5 consecutive weeks. Pre and post intervention scores of Visual analogue scale (VAS), range of movement and Shoulder Pain and Disability Index (SPDI) were recorded. Paired sample t-test was used to analyze the results within groups.

**Results:**

After 5 weeks of intervention both groups made significant improvements in all outcome measures (p < 0.001). Intra group analysis showed no significant difference between two groups (p > 0.05). Mean VAS and SPADI difference was 2.23 and 22 in General exercise & manual therapy group and 2.33 and 23 in General exercise group respectively.

**Conclusion:**

Both exercises with manual therapy and exercises alone are equally effective in the management of adhesive capsulits of the shoulder joint.

## INTRODUCTION

Adhesive capsulitis is one of the most common musculoskeletal complaints which is characterized by loss of both active and passive range of motion. Its prevalence is estimated to be higher than 2 percent and 70 percent victims are women and strikes individual after fifth decade of life.^1^ Adhesive capsulitis of the glenohumeral joint often involves the non-dominant extremity.^2^ Adhesive capsulitis usually results in both shoulders but involving opposite glenohumeral joint encompass after a long time of previous shoulder existence, however certainly not involving the identical glenohumeral joint again.^3^ The documented literature on the prevalence of adhesive capsulitis is very limited and no official research is available in Pakistan.

An Indian study documented prevalence adhesive capsulitis of shoulder between 50 to 70 years of age group. The same study reported diabetes mellitus and monotonous life style as risk factor for the onset of illness.^4^ A study conducted in Bangladesh reported prevalence of adhesive capsulitis in males and females for ratio of 1.8:1 of 11 percent rheumatic disease. The same study defines manual labor and psychiatric conditions as risk factors for the condition.^5^ Adhesive Capsulitis also termed as frozen shoulder is a state that is caused by pain and the limited shoulder range of motion.^6^ The etiology of primary type adhesive capsulitis is yet unidentified. It is related with some other systemic condition such as diabetes mellitus, thyroid disorders or Parkinson‘s disease.^7^,^8^ A detailed study is performed on the histological samples of adhesive capsulitis patients capsular tissue. This study depicted a picture similar to Dupuytren‘s disease. According to the author this happens because of rise in collagen formation, myofibroblasts and fibroplasias. It is notable here, fibro proliferative mechanism had caused 60% of idiopathic adhesive capsulitis in the history of Dupuytren‘s disease.^9^ Management of adhesive capsulitis is a massive challenge and the literature highlighted various types of interventions. These interventions include surgery, steroid injections, oral steroids, electro acupuncture, soft tissue therapy, laser therapy, manipulation under anesthesia, placebo and physical therapy.^10^

A systematic review has suggested that steroid therapy, laser therapy and manual mobilization techniques are effective in the management of adhesive capsulities.^11^ In this review Maitland’s grade I, II & grade III, IV mobilization were evaluated and the overall conclusion supports the use of Maitland’s grade III & IV techniques in the management of adhesive capsulitis.^11^,^12^ Another multiple mobilization treatment study showed that mobilization with movement and end-range mobilization are effective in primary phase Adhesive capsulitis, however no significant effects in secondary adhesive capsultis.^11^,^13^ In contrast mobilization therapy has no significant effects in the management of adhesive capsulitis as compared to steroid therapy.^14^ A randomized controlled trial showed that friction massage and range of movement exercises are more effective as compared to hot pack and short wave diathermy (SWD) in managing adhesive capsulitis.^15^ In another study stretching exercises with heating showed further enhancement in decreasing pain and increasing range of motion in adhesive capsulities.^6^ A few studies have been done to explore the effects of manual therapy and exercises in the management of adhesive capsulitis. However, as to the author’s knowledge no studies have been done to look at whether addition of exercise to specific Maitland’s mobilization will produce any additional beneficial effects. Thus the objective of this study was to compare the Efficacy of General Exercises with and without Maitland’s mobilization Therapy for the management of adhesive capsulitis of the Shoulder.

## METHODS

This randomized experimental study was conducted at institute of physical medicine and rehabilitation Dow university of Health sciences Karachi between January, 2014 and July, 2014 after the institutional review board (IRB) approved the study. Participants of both genders, age ranging from 25 to 60 years were recruited through non-probability purposive sampling technique. The inclusion criteria were One-sided shoulder involvement, Diagnosed patients with complains of pain & shoulder range of movement restriction for more than 3 months according to Reeves classification for adhesive capsulitis.^16^ The exclusion criteria were additional shoulder or cervical pathology, Diabetes Mellitus, Infection, severe trauma of fracture, Pregnancy, carcinoma patients, severe cardiac or psychiatric conditions, Insertion of pace maker and any other serious medical condition that would stop active contribution in the study.

The sample size was 44 participants which were calculated by placing 99 percent power of test and 99 percent confidence interval. PASS software repeated measure of analysis (RM- ANOVA) calculated sample size of 6. By optimizing 20 subjects in a group & after counting 10% drop rate, total of 22 participants in a group got the appreciation. After written informed consent participants were randomly allocated to General Exercise & Manual therapy group (n=22) and General exercise therapy group (n=22) by simple randomization method. After pre intervention assessment participants in General Exercise & Manual therapy group was treated with Maitland mobilization techniques on glenohumeral joint in grade II & III. Techniques applied were postero-anterior, antero-posterior & inferior / caudal glides. In addition the participants performed general shoulder exercises consisting of flexion, abduction stretches, cross over arm stretches, internal and external rotation stretches with and without towel and Codman pendulum exercises.

The general exercises therapy group only received exercises same as the other group. The treatment was applied 3 days in a week for 5 consecutive weeks. Each session lasted 45 minutes including manual mobilization techniques & general exercises. All mobilizations were given in a supine position on the treatment couch. Every glide workout counted in 2 to 3 oscillations in a second for about 30 sec. and providing for 5 sets. Both groups received a home exercise program of same exercises to be performed on daily basis at home. Pre and post treatment evaluation of shoulder pain, range of movement and function were evaluated with visual analogue scale (VAS), goniometry and shoulder pain and disability index.

Version 16.0 of SPSS analyzed the data. Mean, SD- standard deviation, confidence interval of 99% with p-value < 0.05 was considered significant for all comparisons to report the study results. First normality of data was checked then paired sample t-test was used to analyze the results within groups.

## RESULTS

Forty four patients participated in the study and one patient was dropped out due to long institutional distance. There were total 22 participants in general exercise and manual therapy group & 21 participants in general exercise group. The distribution of females and males patients in each group was equal. The mean age of the participants in general exercises and manual therapy group was 51.31 years and for the general exercise group was 51.71 years.

At the end of 5 weeks intervention, mean scores in all outcome measures visual analogue scale (VAS), range of movement and shoulder pain and disability index (SPDI) significantly increased in both groups with p-value<0.05 for VAS, shoulder abduction, external rotation, internal rotation range of movements & SPDI. In General Exercise and Manual Therapy Group mean VAS decreased to 5.45 from 7.68. Shoulder abduction increased to 87.22 from 73.41, external rotation increased to 49.18 from 41.86, internal rotation increased to 62.54 from 53.82. Shoulder disability on SPADI Scale decreased to 56.43 from 78.43. In General Exercise group mean VAS decreased to 5.23 from 7.57. Shoulder abduction increased to 95.43 from 80.33, external rotation increased to 58.66 from 50.95, internal rotation increased to 63.48 from 51.95. Shoulder disability on SPADI Scale decreased to 49.37 from 71.13. (Table I & II)

**Table-I:** Pre and Post results of general exercise and manual therapy group.

Outcome Measure	Pre Values	Post Values	P-Value
VAS	7.68 (1.81)	5.45 (1.53)	< 0.01
ABD.(degree)	73.41 (15.48)	87.22 (21.27)	< 0.01
ER.(degree)	41.86 (16.85)	49.18 (18.09)	< 0.01
IR.(degree)	53.82 (15.93)	62.54 (15.91)	< 0.01
SPADI	78.43 (8.93)	56.43 (11.21)	< 0.01

GEM –general exercises group with mobilization therapy, VAS –Visual analog scale, ABD –Abduction, ER –External rotation, IR –Internal rotation, SPADI –shoulder pain & disability index. ABD, ER, IR were measured in degrees.

**Table-II:** Pre and Post results of general exercise group.

Outcome Measure	Pre Values	Post Values	P-Value
VAS	7.57 (1.47)	5.23 (1.54)	< 0.01
ABD.(degree)	80.33 (19.69)	95.43 (21.72)	< 0.01
ER.(degree)	50.95 (17.63)	58.66 (16.44)	< 0.01
IR.(degree)	51.95 (13.47)	63.48 (16.73)	< 0.01
SPADI	71.13 (14.65)	49.37 (13.96)	< 0.01

GEM –general exercises group with mobilization therapy, VAS –Visual analog scale, ABD –Abduction, ER –External rotation, IR –Internal rotation, SPADI –shoulder pain & disability index. ABD, ER, IR were measured in degrees.

Intra-group analysis showed no statistically significant difference between the two groups in all outcome measures (p-value >0.05). Mean VAS and SPADI difference was 2.23 and 22 in General exercise & manual therapy group and 2.33 and 23 in General exercise group respectively. (Table-III)

**Table-III:** Differences of Pre &Post results between general exercise & manual therapy group & general exercise group.

Outcome Measure	General Exercise & Manual Therapy Group	General Exercise Group	P-Value
VAS	2.23 (1.37)	2.33 (1.46)	0.808
ABD.(degree)	13.81 (12.24)	15.09 (7.68)	0.686
ER.(degree)	7.32 (4.90)	7.71 (4.30)	0.780
IR.(degree)	8.73(5.74)	11.52 (7.48)	0.175
SPADI	22 (8.65)	23 (13.77)	0.790

GEM –general exercises group with mobilization therapy, VAS –Visual analog scale, ABD –Abduction, ER –External rotation, IR –Internal rotation, SPADI –shoulder pain & disability index. ABD, ER, IR were measured in degrees.

## DISCUSSION

This was a comparative study to evaluate the effectiveness of manual mobilization therapy along with general exercises and general exercises alone. This study showed that adhesive capsulitis is more common in females and more than 70% participants were females. The documented burden of adhesive capsulitis in females is around 10% in age ranging from 40-60 years.^2^,^17^ Present study welcomed participants from age 25 to 60 but more than 90% of female population and all male participants were found to be between age range 40-60 years.

Current study reveals that none of the two interventions is superior over one another. Manual mobilization along with exercises and exercises program alone designed for adhesive capsulitis reduced the pain intensity, increased range of motion of shoulder for external rotation, abduction and internal rotation, and SPADI showed improvements in pain and disability level. However, there was no considerable difference between the two groups. A study reported healthy effects of Maitland mobilization techniques with 100 sample size and large intervention duration of 15 weeks in a multicentre study.^11^ These results are in line with present study although current study had short duration of five weeks of intervention and carried out in a single centre population.

In contrast to this study Jugrel *et al*. did not show any significant difference for shoulder active external and internal rotation (p> 0.005).^18^ This study applied a 4 weeks comprehensive rehabilitation program consisting of 10 exercises in pool & gymnasium for half an hour per day, 5 to 10 massage procedures for about 20 minutes in a day & electrical therapy. Insignificant results may be due to the fact that in this study the duration of treatment was 20 minutes whereas in present study 45 minutes exercise sessions were provided hence showed significant difference in outcomes (p<0.001). Chen *et al*. supports our study results and found that Maitland passive mobilization therapy is not more effective than advice and exercises alone for the purpose of reducing shoulder pain and stiffness.^19^ In another two non randomized studies have suggested that Maitland postero-anterior or anterio-posterior glides are effective in improving shoulder external rotation range of movement.^20^,^21^ It is difficult to compare the results of these two case control studies with our study because we included a treatment group and a control group and did not report any significant difference between the two groups. In contrast to present study active physical therapy treatment including stretching and mobilization of shoulder, cervical and thoracic spine as compared to sham ultrasound for 30 minutes failed to show improvements in pain, quality of life and function (p-value > 0.005).^22^ Present study used Maitland mobilizations on glenohumeral joint along with general exercises for duration of 45 minutes with 15 sessions and showed improvements in pain and function. The difference in results may be due to the fact that the above study has also performed mobilization on thoracic and cervical spine whereas we were focused on glenohumeral joint. Secondly the duration of treatment was 30 minutes as compare to our study which was 45 minutes.

Maricar *et al*. has suggested that manual therapy in combination with exercise therapy significantly improved pain and range of movement in adhesive capsulitis.^23^ The duration of this study was 15 weeks which may have caused better results. Furthermore, mobilization with exercises improved shoulder external and internal rotations which were evaluated in standing position through inclinometer.^24^ While in current study, rotational evaluations were made with the patient in supine position with available range of abduction through goniometer. Soderberg *et al*. worked on rotational movements of shoulder in different positions and described major differences in the maximal torque measured. According to them, peak torque is observed in neutral sitting position.^25^ Mix opinions are documented regarding effectiveness of exercises and manual mobilization in the management of adhesive capsulitis which might be due to characteristics of participants, unit centre study, variation in type, frequency, intensity or duration of mobilization and exercise. Future studies may focus on specific type of exercise and mobilization techniques in the management of Adhesive capsulitis.

## CONCLUSION

Both manual mobilization therapy along with general exercises and exercises alone brought improvements in outcome measure scales for pain, glenohumeral ranges and shoulder pain and disability index but none of intervention is significantly effective over one another in 5 weeks of treatment.
